# Bioabsorbable nerve conduits three-dimensionally coated with human induced pluripotent stem cell-derived neural stem/progenitor cells promote peripheral nerve regeneration in rats

**DOI:** 10.1038/s41598-021-83385-9

**Published:** 2021-02-18

**Authors:** Ema Onode, Takuya Uemura, Kiyohito Takamatsu, Takuya Yokoi, Kosuke Shintani, Shunpei Hama, Yusuke Miyashima, Mitsuhiro Okada, Hiroaki Nakamura

**Affiliations:** 1 Department of Orthopaedic Surgery, Osaka City University Graduate School of Medicine, 1-4-3 Asahimachi, Abeno-ku, Osaka, 545-8585 Japan; 2 Department of Orthopaedic Surgery, Osaka General Hospital of West Japan Railway Company, Osaka, Japan; 3 Department of Orthopaedic Surgery, Yodogawa Christian Hospital, Osaka, Japan; 4 Department of Pediatric Orthopaedic Surgery, Osaka City General Hospital, Osaka, Japan

**Keywords:** Neuroscience, Stem cells, Neurology, Engineering, Materials science

## Abstract

Peripheral nerve regeneration using nerve conduits has been less effective than autogenous nerve grafts. To overcome this hurdle, we developed a tissue-engineered nerve conduit coated with mouse induced pluripotent stem cell (iPSC)-derived neurospheres, for the first time, which accelerated nerve regeneration in mice. We previously demonstrated the long-term efficacy and safety outcomes of this hybrid nerve conduit for mouse peripheral nerve regeneration. In this study, we investigated the therapeutic potential of nerve conduits coated with human iPSC (hiPSC)-derived neurospheres in rat sciatic nerve defects, as a translational preclinical study. The hiPSC-derived quaternary neurospheres containing neural stem/progenitor cells were three-dimensionally cultured within the nerve conduit (poly l-lactide and polycaprolactone copolymer) for 14 days. Complete 5-mm defects were created as a small size peripheral nerve defect in sciatic nerves of athymic nude rats and reconstructed with nerve conduit alone (control group), nerve conduits coated with hiPSC-derived neurospheres (iPS group), and autogenous nerve grafts (autograft group) (n = 8 per group). The survival of the iPSC-derived neurospheres was continuously tracked using in vivo imaging. At 12 weeks postoperatively, motor and sensory function and histological nerve regeneration were evaluated. Before implantation, the hiPSC-derived quaternary neurospheres that three-dimensional coated the nerve conduit were differentiated into Schwann-like cells. The transplanted hiPSC-derived neurospheres survived for at least 56 days after implantation. The iPS group showed non-significance higher sensory regeneration than the autograft group. Although there was no actual motor functional nerve regeneration in the three groups: control, iPS, and autograft groups, the motor function in the iPS group recovered significantly better than that in the control group, but it did not recover to the same level as that in the autograft group. Histologically, the iPS group demonstrated significantly higher axon numbers and areas, and lower G-ratio values than the control group, whereas the autograft group demonstrated the highest axon numbers and areas and the lowest G-ratio values. Nerve conduit three-dimensionally coated with hiPSC-derived neurospheres promoted axonal regeneration and functional recovery in repairing rat sciatic nerve small size defects. Transplantation of hiPSC-derived neurospheres with nerve conduits is a promising clinical iPSC-based cell therapy for the treatment of peripheral nerve defects.

## Introduction

Autogenous nerve grafts are still considered the gold standard treatment for repairing peripheral nerve defects; however, they involve donor site morbidity accompanied by sacrificing the other intact peripheral nerves^1,2^. In recent years, various kinds of bioabsorbable nerve conduits have become available for repairing peripheral nerve segmental defects, without sacrificing the intact nerves^3–13^. However, nerve regeneration using these nerve conduits has been less efficacious than using autogenous nerve grafts, as some authors advocated^1,14–16^. To overcome this hurdle, numerous modifications of the addition of supportive cells and/or growth factors to the nerve conduits, as a scaffold, have been attempted in animal models^6,7,13,17,18^. Although it has been reported that hybrid nerve conduits coated with supportive cells, such as Schwann cells, adipose-derived stem cells, and bone marrow-derived stem cells and/or growth factors such as fibroblast growth factor and nerve growth factor, enhanced the axonal regeneration, these hybrid nerve conduits are not yet clinically available^13,19–25^.

We previously developed a tissue-engineered bioabsorbable nerve conduit coated with mouse induced pluripotent stem cell (iPSC)-derived neurospheres containing neural stem/progenitor cells that accelerated axonal regeneration and functional recovery in repairing sciatic nerve defects in both young and aged mice^26–29^. Furthermore, we revealed the long-term efficacy and safety outcomes of transplantation of the mouse iPSC-derived neurospheres with nerve conduits for peripheral nerve regeneration in mice^30^. To initiate the first-in-human clinical application of iPSC-based cell therapy of peripheral nerves with nerve conduits, it is essential to confirm the efficacy of the human iPSC (hiPSC)-derived neurospheres with nerve conduits, switching from mouse iPSCs to preclinical research. In the present study, we evaluated the efficacy of nerve conduits coated with hiPSC-derived neurospheres in comparison with autologous nerve grafts in the sciatic nerve defects of immunosuppressed rats.

## Methods

### Neural induction of iPSCs and lentivirus transduction

The primary neurospheres containing neural stem/progenitor cells, derived from the hiPSCs (201B7), were identical to those used in previous reports on the therapeutic potential of hiPSCs for spinal cord injury models and were kindly provided by Dr. Okano and Dr. Nakamura (Keio University School of Medicine)^31–35^. The culture and neural induction of hiPSC were also performed with the same procedure as previously described^33,34,36–38^. Briefly, the primary neurospheres were passaged into secondary, tertiary, and quaternary neurospheres, which were used for transplantation. According to the same procedures as described previously, the hiPSC-derived quaternary neurospheres were dissociated and infected with lentivirus expressing *ff*Luc, a green fluorescent protein (modified from Venus) fused to a luminescence protein (Luciferase 2), under control of the EF promoter (pCSII-EF-Venus) for bioluminescent image tracing of grafted neurospheres^38–40^.

### Nerve conduit

**Figure 1:**
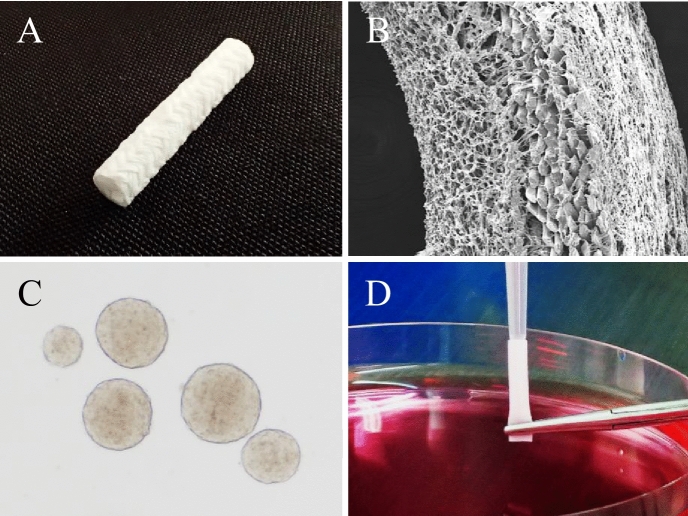
(**A**) Gross appearance of the bioabsorbable nerve conduit. (**B**) Scanning electron microscope image of a cross-section of the two-layered conduit; the inner layer has a honeycomb structure that functions as a scaffold. (**C**) Neurospheres derived from the hiPSCs. (**D**) Seeding of the hiPSC-derived neurospheres into the nerve conduit in vitro.

The bioabsorbable polymer nerve conduit (outer diameter: 3 mm; inner diameter: 2 mm; length: 7 mm) used in the present study was identical to models used in our previous studies on the treatment of peripheral nerve defects (Fig. 1)^26–30,41,42^. The nerve conduit consists of two layers: the outer layer, composed of a poly l-lactide (PLA) multifilament fiber mesh; and the inner layer, composed of a 50% PLA and 50% polycaprolactone (PCL) porous sponge (Fig. 1)^43,44^. The nerve conduit was elastic enough to maintain its tubular structure during future axonal growth, but flexible enough to allow easy handling. In particular, the PLA and PCL copolymer sponge of the inner layer has a honeycomb structure with pores of 20–400 µm, into which regeneration-facilitating cells, such as Schwann cells and iPSC-derived neurospheres, could enter and proliferate as a scaffold^26–30,41,45^.

### Preparation of nerve conduits three-dimensionally coated with hiPSC-derived neurospheres

Nerve conduits coated with hiPSC-derived neurospheres were prepared according to the same procedures as described in our previous reports^26–30^. Briefly, the day-12 hiPSC-derived quaternary neurospheres were labeled with lentivirus for expressing *ff*Luc and then dissociated into single cells, using TrypLE select (Gibco, Tokyo, Japan). After prewetting the conduit with 70% ethanol and rinsing with physiological saline, one end of the nerve conduit was carefully infused with 50 µL suspension medium, which was just filled in the nerve conduit, including 4.0 × 10^6^ cells, while the other end the nerve conduit was clamped with forceps to avoid the suspension from flowing out of the conduit. (Fig. 1)^26–30^. Then, nerve conduits were placed and incubated in KBM Neural Stem Cell medium (Kohjin Bio, Saitama, Japan), supplemented with MACS NeuroBrew-21 (Miltenyi Biotec, Bergisch Gladbach, Germany), Leukemia Inhibitory Factor human (Merck, Darmstadt, Germany), and recombinant human FGF-basic (Pepro Tech, Rocky Hill, New Jersey, USA) for 14 days. This completed the process of three-dimensional coating the nerve conduits with hiPSC-derived quaternary neurospheres. Before implantation, nerve conduits, coated with hiPSC-derived quaternary neurospheres, were histologically evaluated to confirm their differentiation into Schwann-like cells, which were the most important for axonal regeneration^26,30^. The nerve conduits were fixed with 4% paraformaldehyde at 4 °C for 24 h and embedded in paraffin. Transverse and longitudinal sections (4 µm thick) of the central part of the nerve conduits were stained with hematoxylin and eosin (HE), and anti-S-100 antibody (Abcam, Cambridge, UK) as a marker of mature Schwann cells for immunohistochemistry.

### Experimental groups and repair of sciatic nerve defects in rats

**Figure 2:**
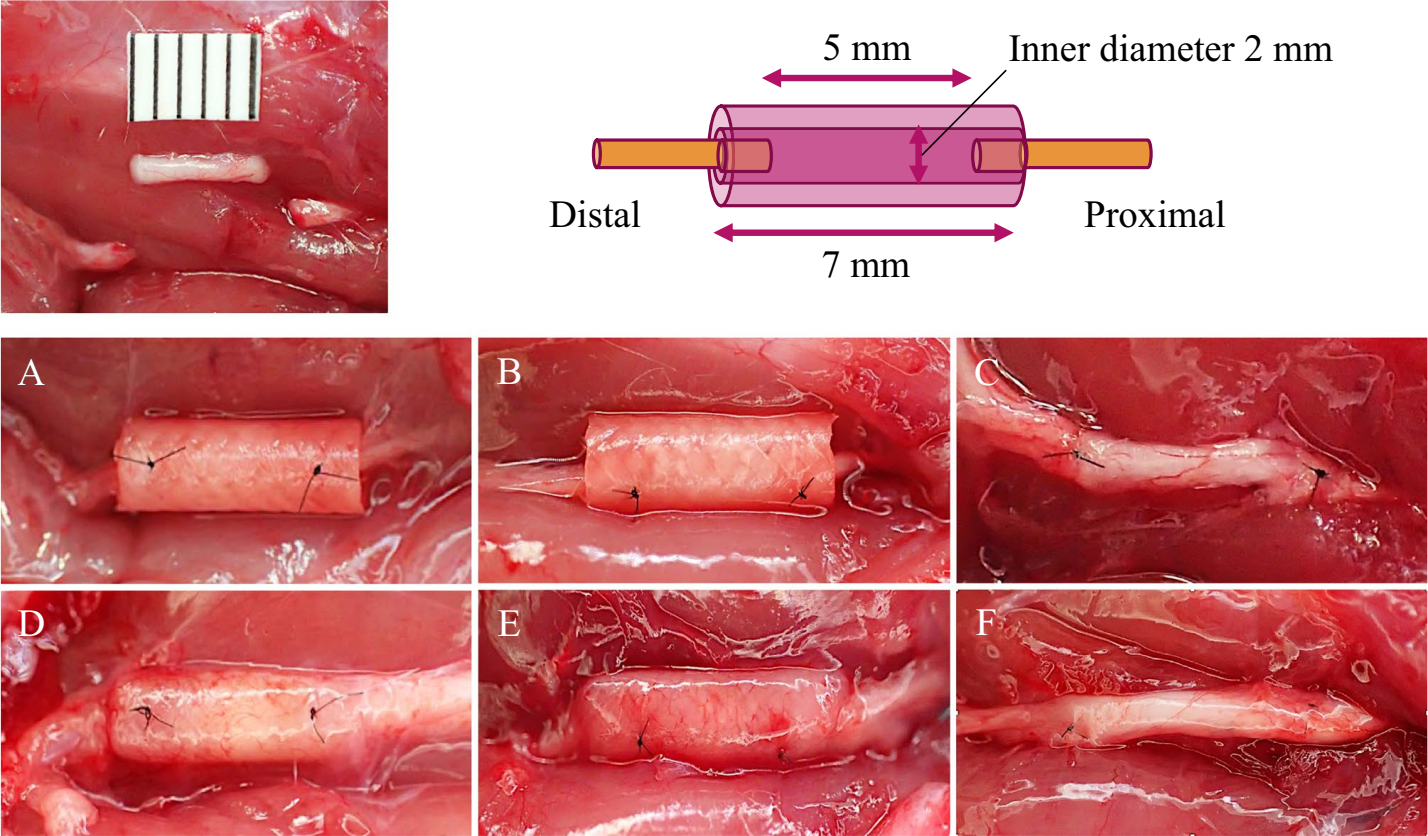
Intraoperative gross findings in the rat sciatic nerve transection experimental model. Reconstructed with the nerve conduit alone (control group, **A** and **D**), the nerve conduit coated with hiPSC-derived neurospheres (iPS group, **B** and **E**), and the autogenous nerve grafts (autograft group, **C** and **F**) at the time of the implantation (**A**–**C**), and 12 weeks after implantation (**D**–**F**).

In total, 28 male athymic nude rats (F344/NJcl-rnu/rnu) aged 8 weeks were purchased from CLEA Japan (Tokyo, Japan), and housed in an air-conditioned room with free access to food and water^46^. All experiments were conducted in strict accordance with the Institutional Guideline for the Care and Use of Laboratory Animals of Osaka City University. After the left sciatic nerve was exposed under anesthetic, 1.15 mL of ketamine (50 mg/mL) and 0.35 mL of 2% xylazine was subcutaneous injected into the right dorsal back; complete 5-mm defects were made as a small size peripheral nerve defect in the left sciatic nerve and reconstructed with nerve conduits or autogenous nerve grafts (Fig. 2)^18^. A total of 24 rats were randomized into three experimental groups as follows: (1) conduits alone without cells (control group); (2) conduits coated with hiPSC-derived neurospheres (iPS group); (3) autogenous nerve grafts (autograft group) (n = 8 per group). In the control and iPS groups, 1-mm lengths of both proximal and distal stumps of the sciatic nerve were pulled into the nerve conduit, and nerve ends were sutured at two proximal and distal locations to the lumen wall with 9-0 nylon sutures in a horizontal mattress pattern under the microscope^26–30^. In the autograft group, the 5-mm defects were reconstructed using the resected nerve itself. The resected nerve was rotated 180°, inserted between the two nerve stumps, and sutured under the microscope with 9–0 nylon sutures (Fig. 2).

### Bioluminescent imaging analysis

The other four rats, which were separated from 24 rats (eight rats in each group), were randomized into two experimental groups for bioluminescent imaging analysis: conduits coated with hiPSC-derived neurospheres labeled with *ff*Luc (n = 3) and conduit coated with hiPSC-derived neurospheres labeled without *ff*Luc as a control (n = 1). Survival of hiPSC-derived neurospheres in the nerve conduits was continuously traced using in vivo analysis. A luciferase substrate, D-luciferin (Perkin Elmer, Waltham, Massachusetts, USA) was directly infused (50 μL) into the nerve conduit, after exploring the nerve conduit under anesthetic, by subcutaneous injection of ketamine and xylazine, and the *ff*Luc-labelled cells were monitored using an In vivo Imaging System (IVIS) spectrum and CCD optical macroscopic imaging system (Perkin Elmer, Waltham, Massachusetts, USA). Bioluminescence signals were observed with luminescent imaging mode (exposure time, 1 min; field of view, 5 cm square) were measured at 4, 7, 14, 28, 56, and 84 days after transplantation. The survival rate of hiPSC-derived neurospheres was quantified by setting the amount of luminescence on the day of transplantation (day 0) to 100 and expressing the increase or decrease as a percentage.

### Evaluation of functional recovery

The recovery of sensory and motor function of each rat’s hind limb (n = 8 per group, total 24 rats) was evaluated at 4, 8, and 12 weeks after repair of the peripheral nerve gaps. The recovery of sensory function was assessed with the manual von Frey filament test, as described previously^47,48^. Briefly, rats were individually placed in small cages on a metal mesh flooring that allowed access to the planter surface of each hind paw. A series of von Frey calibrated filaments was gently applied perpendicular to the planter surface of the ipsilateral or contralateral hind paw (avoiding the toe pads) until the filament flexed, then the filament was held in place for three seconds. The nociceptive threshold was expressed as the force at which the rat withdrew the paw in response to stimulus. A cutoff value was set at a force at which the stimulus lifted the paw without response. The ipsilateral/ contralateral ratio was calculated using the von Frey filament test.

The recovery of motor function was assessed with walking track analysis using a sciatic functional index (SFI) at 4, 8, and 12 weeks postoperatively. As described in previous reports, rats walked up an inclined path with inked paws, and their footprints were recorded on a white paper^17,40,48,49^. The measurements of toe spread and print length were made on the experimental side: experimental toe spread (ETS), experimental print length (EPL), contralateral control side, normal toe spread (NTS), and normal print length (NPL). SFI was calculated from the values of ETS, NTS, EPL, and NPL as follows: SFI = 118.9 × ([ETS − NTS]/NTS) − 51.2 × ([EPL − NPL]/NPL) − 7.5. The SFI value, which is close to 0, indicates normal nerve function. Mean values calculated from three print-length measurements obtained from each limb were taken from every rat, and subsequent mean values of SFI were then calculated for each group.

The recovery of motor function of each mouse’s hind limb was also assessed at 12 weeks postoperatively on the basis of electrophysiological recordings from the sciatic nerve as described previously^29,42,50^. Briefly, mice were anesthetized and the bilateral sciatic nerves were carefully exposed. Electrophysiological signals were obtained using a NICOLET VIKING SELECT electromyogram machine (Natus Neurology Inc., Wisconsin, US). The nerve at the proximal end of the nerve conduit was stimulated with a monopolar 28-G needle electrode in both the control and iPS groups. The nerve proximal to the suture point was stimulated with a monopolar 28-G needle electrode in the autograft group. The recording needle electrode was placed in the gastrocnemius muscle. The reference needle electrode was placed at the Achilles tendon. A series of nerve stimulations were performed with repetitively generated single pulses of 0.1 ms duration until a maximal artifact-free compound muscle action potential (CMAP) motor response was evoked. CMAP amplitudes were recorded at three different recording locations to optimize the recording conditions. CMAP amplitude ratios of experimental to unaffected side are reported.

To evaluate the functional recovery of the sciatic nerve, the gastrocnemius muscles on both the affected and unaffected sides were resected and their wet weights were measured (n = 8 per group, total 24 rats) at 12 weeks postoperatively. The specimens were immersed in 4% paraformaldehyde overnight and embedded in paraffin. Five-micrometer-thick transverse sections at the level of the largest area of the muscle were stained with hematoxylin and eosin to evaluate muscle atrophy.

### Histological evaluation and histomorphometry

Twelve weeks after repair of the peripheral nerve gaps, the nerve conduits and grafted nerves were harvested (n = 8 per group, total 24 rats), and fixed in 4% paraformaldehyde overnight at 4 °C then embedded in paraffin. Axonal regeneration was examined in central transverse sections with immunohistochemistry using anti-neurofilament protein antibodies (Millipore, Temecula, CA, USA)^27–30,42^. For each nerve conduit and grafted nerve, an image showing the greatest number of regenerated axons was photographed at 400× magnification with an Olympus DP74 camera (Olympus corporation, Tokyo, Japan). Then, the numbers and areas of regenerating axons that were positive for neurofilament protein from five of the randomly selected fields in the image were calculated automatically using ImageJ software (National Institutes of Health)^29,42^.

Electron microscopic histomorphological analyses were performed in the control, iPS, and autograft groups (n = 3 per group) 12 weeks postoperatively. Briefly, the nerve conduits and grafted nerves were prefixed with 2.5% glutaraldehyde and 2% paraformaldehyde, followed by immersion in 1% osmium tetroxide. Samples were dehydrated using serial ethanol dilutions (50–100% ethanol) and impregnated with 100% epoxy resin. Semi-thin (1-mm) sections at the midpoint of the nerve conduit or grafted nerve were cut and stained with toluidine blue; ultra-thin (70-nm) sections of epoxy resin-embedded tissues were subsequently obtained. These sections were stained with 5% uranyl acetate, with 50% ethanol and Reynolds’ lead citrate, and examined using a transmission electron microscope (Talos F200C G2, Thermo Fisher Scientific). Twenty myelinated axons of each nerve were randomly selected, and G-ratio values (axon perimeter/ fiber perimeter) were measured using ImageJ software (National Institutes of Health)^17,40^. The lower the G-ratio, the higher the degree of remyelination.

### Ethics statement

All experiments involving animal subjects were carried out in accordance with the institutional guidelines and regulations for animal research. All experimental protocols and ethical permission were approved by our institutional licensing research committee. The approval number is 10025: Osaka City University Research Control Centre.

### Statistical analysis

All experimental data are expressed as the mean ± standard deviation (SD). One-way ANOVA with Fisher’s test were performed using StatPlus software (AnalystSoft Inc., Walnut, CA, USA). *p* < 0.05 denoted statistical significance.

## Results

### Nerve conduits three-dimensionally coated with hiPSC-derived neurospheres

**Figure 3:**
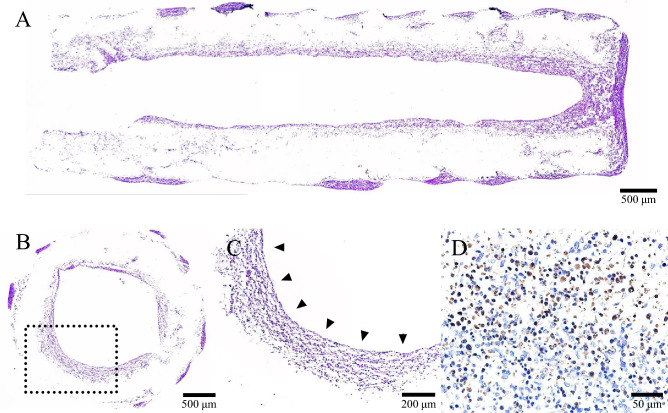
HE-stained images in a longitudinal (**A**) and transverse (**B** and **C**) sections of the central part of the nerve conduits after 14 days of coating with hiPSC-derived quaternary neurospheres prior to implantation. (**C**) A higher magnification of the region of inner porous sponge within the nerve conduit depicted in (**B**). The hiPSC-derived quaternary neurospheres have adhered to the inner surface of the nerve conduits (black triangles) and have migrated into the inner porous sponge. (**D**) Immunohistochemically stained image with anti-S-100 antibody.

The hiPSC-derived quaternary neurospheres had adhered uniformly and tightly to the inner surface of the nerve conduits and had migrated into the inner porous sponge in the HE-stained images (Fig. 3). The engrafted cells on the nerve conduits were positive for anti-S-100 antibody by immunohistochemistry. This indicated that hiPSC-derived quaternary neurospheres coating the nerve conduit could be differentiated into Schwann-like cells; these hiPSC-derived Schwann-like cells could then be three-dimensional-cultured in the nerve conduits as a scaffold.

### Survival of transplanted hiPSC-derived neurospheres

**Figure 4:**
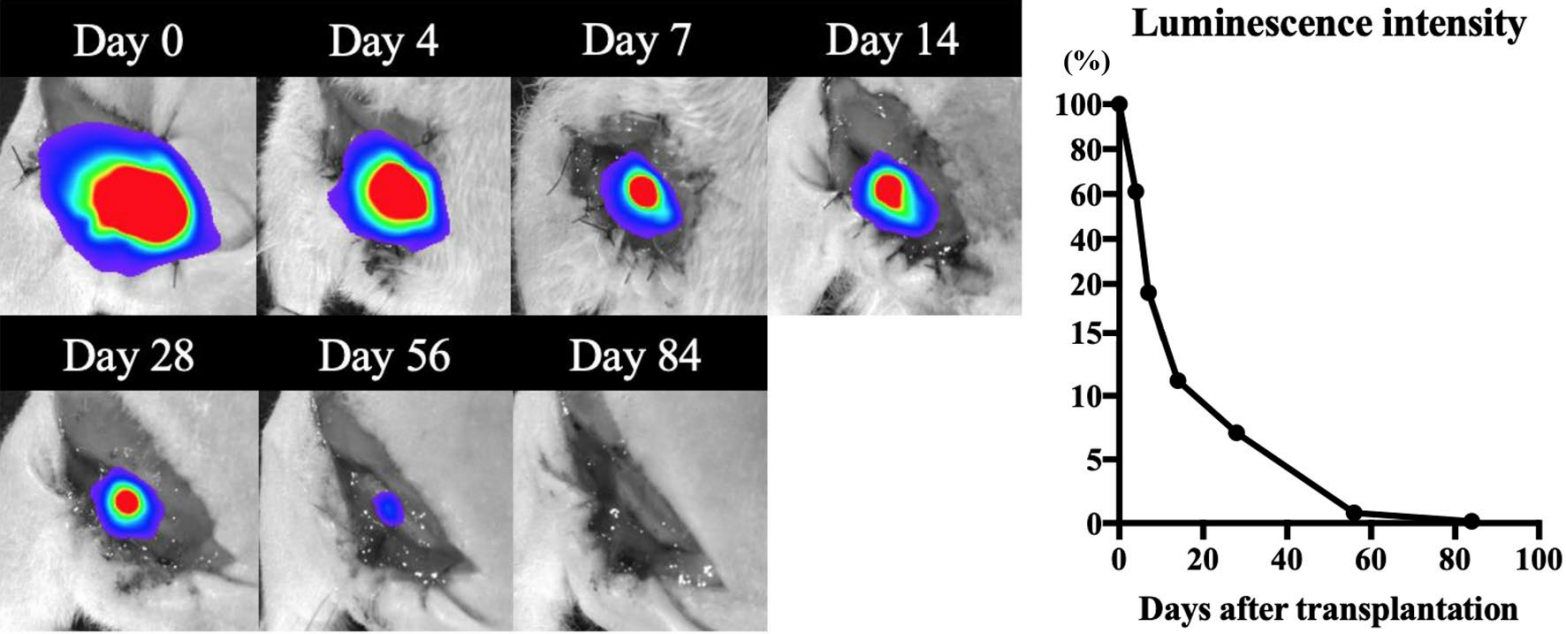
Images and quantitative analyses of the photon counts in the in vivo imaging system. The percentage of luminescence intensity was 61.2, 16.1, 11.2, 7.1, 0.8, and 0.2 at 4, 7, 14, 28, 56, and 84 days after implantation, respectively.

Among four rats, two rats in which conduits coated with hiPSC-derived neurospheres labeled with *ff*Luc, died before 14 days. The mean percentage of survival of the transplanted hiPSC-derived neurospheres was 61.2 ± 20.8, 16.1 ± 4.5, 11.2 ± 0, 7.1 ± 0, 0.8 ± 0, and 0.2 ± 0 at 4, 7, 14, 28, 56, and 84 days after implantation, respectively (Fig. 4). Cell tracing in IVIS showed that the grafted hiPSC-derived neurospheres with nerve conduits decreased gradually after implantation. Although they consistently survived for at least 56 days, they eventually disappeared after 84 days.

### Functional recovery

**Figure 5:**
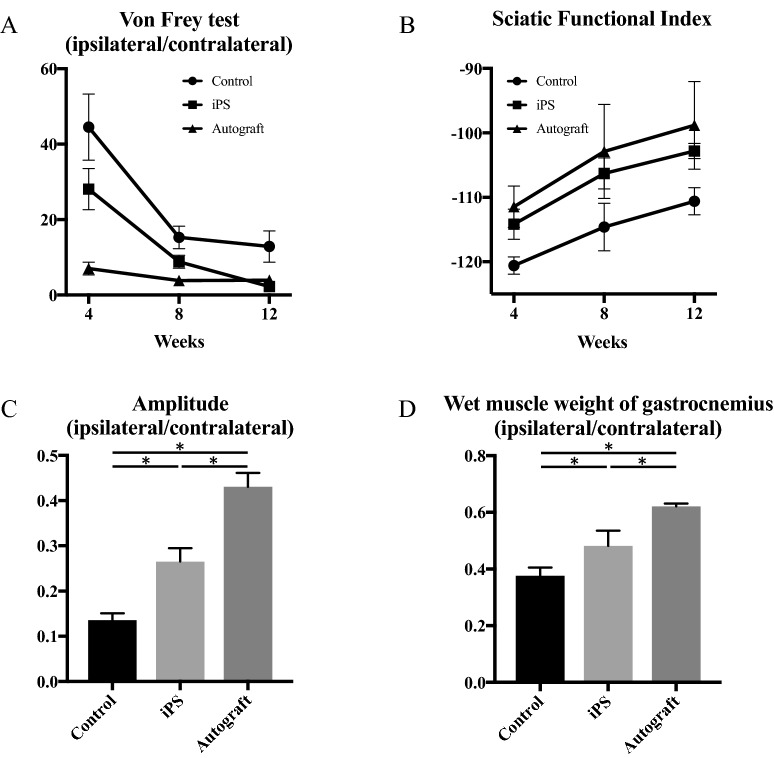

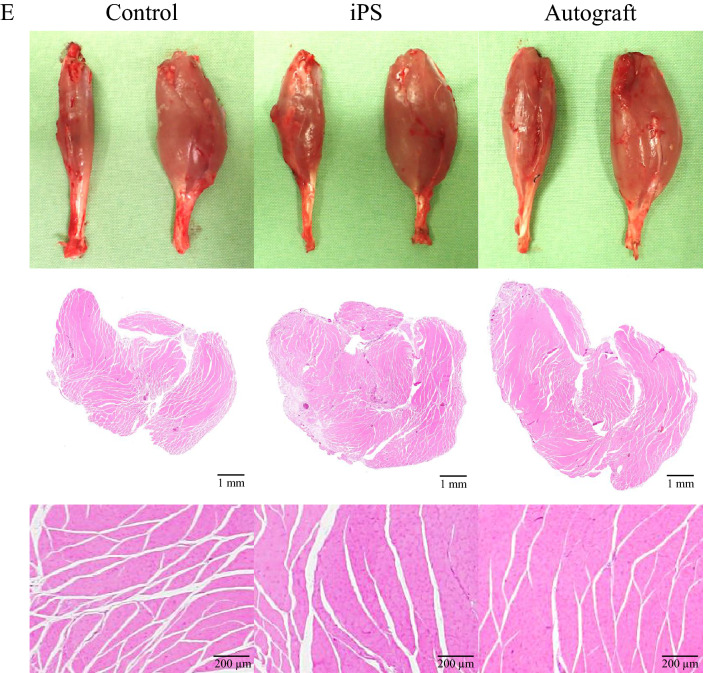
Sensory function assessed with the manual von Frey filament test (**A**) and motor function assessed with SFI of walking track analysis (**B**), amplitude of the CMAP of the gastrocnemius muscles (**C**), and wet muscle weight of the gastrocnemius (**D**) postoperatively. Representative gross appearances and HE-stained images of the gastrocnemius muscle (**E**). (**A**) Control group: 44.5 ± 8.8 (vs. iPS group: p = 0.07, vs. autograft group: p < 0.01), iPS group: 28.1 ± 5.5 (vs. autograft group: p = 0.02), autograft group: 7.0 ± 1.7 at 4 weeks, control group: 15.3 ± 3.0 (vs. iPS group: p = 0.04, vs. autograft group: p < 0.01), iPS group: 8.8 ± 1.7 (vs. autograft group: p = 0.10), autograft group: 3.8 ± 0.7 at 8 weeks, control group: 12.9 ± 4.2 (vs. iPS group: p = 0.01, vs. autograft group: p = 0.02), iPS group: 2.2 ± 0.4 (vs. autograft group: p = 0.63), autograft group: 3.9 ± 0.7 at 12 weeks after transplantation. *p < 0.05. (**B**) Control group: − 120.6 ± 1.3 (vs. iPS group: p < 0.01, vs. autograft group: p < 0.01), iPS group: − 114.2 ± 2.3 (vs. autograft group: p = 0.05), autograft group: − 111.5 ± 3.2 at 4 weeks, control group: − 114.6 ± 3.7 (vs. iPS group: p < 0.01, vs. autograft group: p < 0.01), iPS group: − 106.3 ± 2.4 (vs. autograft group: p = 0.21), autograft group: − 102.9 ± 7.3 at 8 weeks, control group: − 110.6 ± 2.1 (vs. iPS group: p < 0.01, vs. autograft group: p < 0.01), iPS group: − 102.8 ± 1.2 (vs. autograft group: p = 0.08), autograft group: − 98.8 ± 6.8 at 12 weeks after transplantation. (**C**) Control group: 0.14 ± 0.02 (vs. iPS group: p < 0.01, vs. autograft group: p < 0.01), iPS group: 0.27 ± 0.03 (vs. autograft group: p < 0.01), autograft group: 0.43 ± 0.03. (**D**) Control group: 0.38 ± 0.03 (vs. iPS group: p = 0.04, vs. autograft group: p < 0.01), iPS group: 0.48 ± 0.05 (vs. autograft group: p < 0.01), autograft group: 0.62 ± 0.01.

The recovery of hind limb sensory and motor function of rats is shown in Fig. 5. In the recovery of sensory function at 12 weeks postoperatively, assessed with the von Frey filament test, the ipsilateral/contralateral ratio of the hind paw withdrawal threshold in the control group (12.9 ± 4.2) was the highest, while those in the iPS (2.2 ± 0.4, p < 0.01) and autograft groups (3.9 ± 0.7, p = 0.02) were significantly lower than in the control group. The ipsilateral/contralateral ratio of the hind paw withdrawal threshold in the iPS group (2.2 ± 0.4, p = 0.63) was non-significantly lower than that in the autograft group (3.9 ± 0.7).

In the recovery of motor function at 12 weeks postoperatively, assessed with walking track analysis, the mean values of the SFI in the iPS (− 102.8 ± 1.2) and autograft groups (− 98.8 ± 6.8) were equal to one another (p = 0.08), but they (iPS: − 102.8 ± 1.2, p < 0.01, autograft: − 98.8 ± 6.8, p < 0.01) were significantly lower compared to the control group (− 110.6 ± 2.1). The aforementioned figures indicated there was no actual motor functional nerve regeneration in the three groups: control, iPS, and autograft groups.

The ipsilateral/contralateral ratio of the amplitude of the CMAP of the gastrocnemius muscles in the autograft group (0.43 ± 0.03) was significantly higher than that in the control (0.14 ± 0.02, p < 0.01) and iPS groups (0.27 ± 0.03, p < 0.01), while the ratio in the iPS group (0.27 ± 0.03, p < 0.01) was significantly higher than that in the control group (0.14 ± 0.02).

Representative gross appearances and histological sections of the gastrocnemius muscle are shown in Fig. 5. The gastrocnemius muscles on the affected side were atrophied in the order of the control, iPS, and autograft groups. The wet muscle weights of the gastrocnemius in the autograft group (0.62 ± 0.01) were significantly greater than those in the control (0.38 ± 0.03, p < 0.01) and iPS (0.48 ± 0.05, p < 0.01) groups, while the weights in the iPS group (0.48 ± 0.05, p = 0.04) were significantly higher than those in the control group (0.38 ± 0.03).

These results indicate that the sensory function in the iPS group could recover up to the same level as the autograft group, and the motor function in the iPS group recovered significantly better than that in the control group, but it did not recover to the same level as that in the autograft group.

### Axon regeneration and myelination

**Figure 6:**
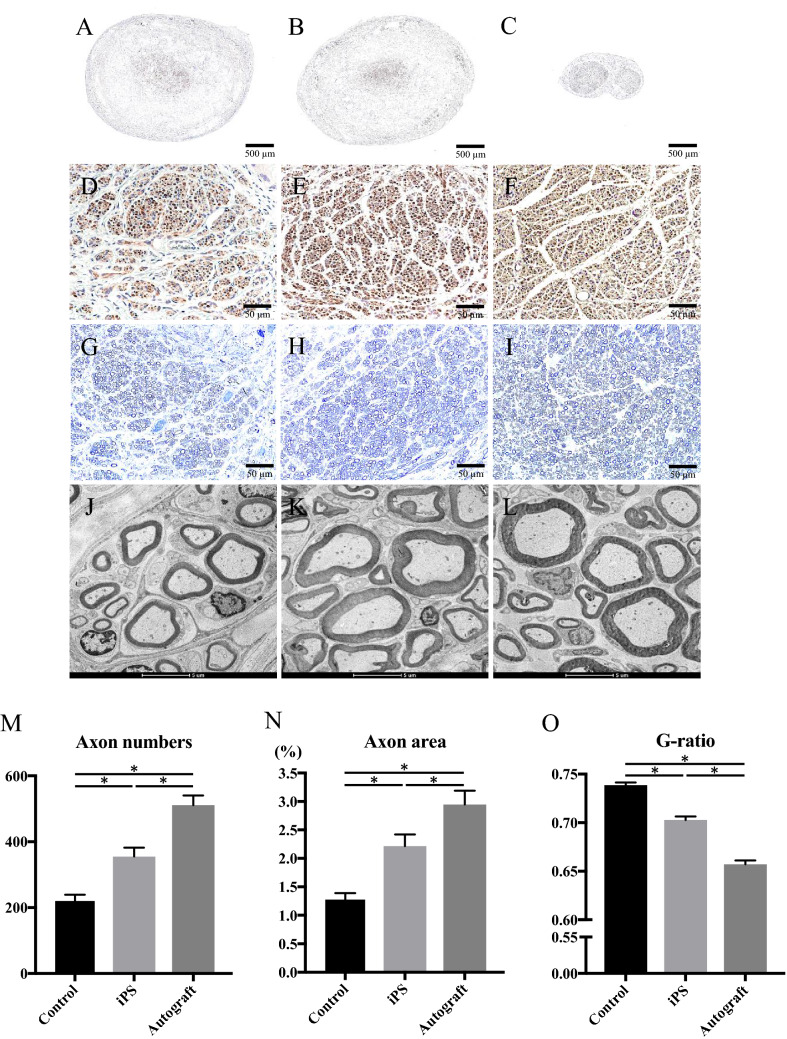
Representative optical microscope (**A**–**I**) and transmission electron microscope (**J**–**L**) images in the central transverse sections of the nerve conduits and grafted nerves 12 weeks after implantation. Immunohistochemistry for neurofilament protein antibody (**A**–**F**), and toluidine blue staining (**G**–**I**). (**A**, **D**, **G**, **J**) The control group. (**B**, **E**, **H**, **K**) The iPS group. (**C**, **F**, **I**, **L**) The autograft group. Axon numbers (**M**), axon areas (**N**), and the G-ratio (**O**) 12 weeks after implantation. *p < 0.05. (**M**) Control group: 220 ± 19 (vs. iPS group: p < 0.01, vs. autograft group: p < 0.01), iPS group: 355 ± 27 (vs. autograft group: p < 0.01), autograft group: 511 ± 30. (**N**) Control group: 1.3 ± 0.1 (vs. iPS group: p < 0.01, vs. autograft group: p < 0.01), iPS group: 2.2 ± 0.2 (vs. autograft group: p = 0.01), autograft group: 2.9 ± 0.2. (**O**) Control group: 0.74 ± 0.003 (vs. iPS group: p < 0.01, vs. autograft group: p < 0.01), iPS group: 0.70 ± 0.004 (vs. autograft group: p < 0.01), autograft group: 0.66 ± 0.004.

Twelve weeks after implantation, the autogenous nerve grafts remained continuous between the nerve stumps, and the lumen structure of the nerve conduits in the control and iPS groups were maintained without collapse, despite the occurrence of bioabsorption (Fig. 2). Histologically, on cross-sections at the midpoint of the nerve repair site, the luminal structure of nerve conduits were also maintained at 12 weeks; regenerating axons, which were positive for anti-neurofilament protein and myelinations, assessed with toluidine blue staining were found in the center of the nerve conduits in both the control and iPS groups (Fig. 6). Specifically, the iPS group demonstrated significantly higher axon numbers (355 ± 27, p < 0.01) and areas (2.2 ± 0.2%, p < 0.01) than the control group (Axon numbers: 220 ± 19, axon areas: 1.3 ± 0.1%), whereas the autograft group demonstrated the highest axon numbers (511 ± 30, p < 0.01) and areas (2.9 ± 0.2%, p < 0.01) among the control, iPS, and autograft groups. The mean values of the G-ratio, showing the degree of myelination, were significantly lower in the iPS group (0.70 ± 0.004, p < 0.01) than in the control group (0.74 ± 0.003), while the lowest values were observed in the autograft group (0.66 ± 0.004, p < 0.01). Thus, coating the nerve conduits with hiPSC-derived quaternary neurospheres accelerated the axon regeneration and myelination in repairing sciatic nerve defects with nerve conduits in rats.

## Discussion

In the present study, nerve conduit three-dimensionally coated with hiPSC-derived neurospheres promoted axonal regeneration and functional recovery in repairing sciatic nerve small size defects in athymic rats, while the transplanted hiPSC-derived neurospheres, as Schwann-like cells, survived for at least 56 days.

We have developed and evaluated this new nerve conduit consisting of PLA and PCL in many basic animal studies for clinical commercial application for peripheral nerve regeneration. We believe that the nerve conduit should be elastic enough to maintain its tubular structure during axonal growth, but flexible enough to allow easy handling during operation. This nerve conduit met the requirements. The elasticity of the tubular structure was secured because of the stiffness of the outer layer PLA multifilament fiber mesh, whose biodegeneration via hydrolysis was slower than that of the inner layer. Flexibility of the tubular structure was conferred by the inner layer of the PLA and PCL copolymer sponge. Moreover, the PLA and PCL copolymer sponge of the inner layer had a honeycomb structure with pores measuring 20–400 µm, in which regeneration-facilitating cells could enter^26–30,41,45^. Based on these merits, we used the nerve conduit in the present study.

The basic animal research of iPSC-based cell therapy for peripheral nerve regeneration with nerve conduits has previously been reported^40,51–54^. Wang et al. demonstrated that the transplanted neural crest stem cells derived from a combination of hiPSCs and human embryonic stem cells (hESCs) with nanofibrous nerve conduits promoted sciatic nerve regeneration in athymic rats^51^. A combination of tubular nerve conduits sustained these hiPSC/hESC-derived neural crest stem cells, and, together with low-intensity pulsed ultrasound, also promoted the regeneration of rat transected sciatic nerve^52^. Moreover, these hiPSC/hESC-derived neural crest stem cells in combination with low-intensity pulsed ultrasound and growth differentiation factor-5 promoted sciatic nerve regeneration and functional recovery in rats^54^. Huang et al. found that the similar integration-free hiPSC-derived neural crest stem cell-seeded nerve conduit accelerated electrophysiological and functional recovery in rats^53^. Additionally, Kimura et al. revealed that the silicone tube seeded stem cells, purified from hiPSC-derived neural crest-like cells, promoted axonal regrowth and remyelination in a murine peripheral nerve defect model^40^. Thus, hiPSC-derived neural crest stem cells, with nerve conduits, have been used in previous studies regarding iPSC-based peripheral nerve regeneration, while the iPSC-derived neurospheres containing neural stem/progenitor cells have been used in our previous studies, including the present study^26–30^.

Although it remains unclear as to which cellular source is effective for peripheral nerve regeneration with nerve conduit, we have consistently used the safe and well-established hiPSC-derived neurospheres containing neural stem/progenitor cells from among a number of supportive cellular sources involving various stem cells. These hiPSC-derived neurospheres have been the most strictly evaluated in numerous translational preclinical studies for the first-in human clinical trial of the iPSC-based cell therapy for spinal cord injury^35,55^. Cell transplantation therapies using iPSCs have progressed greatly in various fields of regenerative medicine since the emergence of iPSCs in 2006^56,57^. Specifically in the field of spinal cord injury from basic animal and laboratory studies to preclinical studies, numerous research projects using the same iPSC-derived neurospheres used in this study have been undertaken and yielded successful results, establishing a safe neural induction protocol from the identified safe iPSCs^33–35,55,58^. Tsuji et al. first reported the transplantation of safe mouse iPSC-derived neurospheres, identical to those used in our previous reports, into the murine spinal cord injury promoted functional recovery without any tumor formation^38^. Next, it was demonstrated that the transplanted hiPSC-derived neurospheres, identical to those used here, promoted motor functional recovery after spinal cord injury in NOD/SCID mice^33^. Then, transplantation of the hiPSC-derived neurospheres was shown to promote axonal regrowth and functional recovery after spinal cord injury in the common marmoset, without tumorigenicity^34^. Finally, based on the efficacy of the results in the aforementioned animal models, a clinical trial for transplantation of the safe hiPSC-derived neurospheres into subacute spinal cord injury patients was recently conducted in Japan, using iPSC stocks matching the patient’s HLA type as allografts instead of the patient’s own cells^55,58^. Following similar logical steps in the field of peripheral nerve injury preclinical applications, the efficacy of the nerve conduits coated with hiPSC-derived neurospheres was verified in the present study and will be evaluated using primate models in the future. Once again, the present study has significant advantages of translational research in peripheral nerve regeneration with nerve conduits, using the most strictly evaluated, established, and advanced hiPSC-derived neurospheres.

In the present study, complete 5-mm defects as a small size defect in immunosuppressed athymic rats were repaired with nerve conduits coated with hiPSC-derived neurospheres. In our previous studies, complete 5-mm defects in non-immunosuppressed mice were repaired with nerve conduits coated with mouse iPSC-derived neurospheres. Therefore, the functional recovery in the present study was better than that in previous studies. Moreover, in the present study, we intentionally used complete 5-mm defects as a small size peripheral nerve defect in athymic rats, although 10-mm defects in rat sciatic nerves have been considered longer gap defects. The reasons for this are as follows. The purpose of the present study was not to promote peripheral nerve regeneration exceeding a large gap defect in rat sciatic nerve, but to prove the efficacy of additional iPSC-derived neurospheres to the nerve conduit in a small size defect as a translational preclinical study. Clinically, the nerve conduit has been applied for only a small defect. We would like to expand the clinical application of nerve conduit in a small size peripheral nerve defect using a nerve conduit coated with iPSC-derived neurospheres. Moreover, the 8-week-old male athymic nude rats (F344/NJcl-rnu/rnu; 175 g) used in the present study were much smaller than immune-responsive common rats (for example, SD rat: 270 g).

In this study, grafted hiPSC-derived neurospheres survived consistently for more than eight weeks, but completely disappeared within 12 weeks due to immune rejection of the xenograft. This was due to the fact that although the athymic nude rats were T-cell deficient, immune rejection through natural killer cells, macrophages, and humoral immunity was possible^46^. Although it remains unclear how the grafted hiPSC-derived neurospheres promoted axonal regeneration, we speculate that the grafted hiPSC-derived neurospheres, which partially differentiated into Schwann-like cells, contribute to enhancing axonal regrowth, indirectly by secreting multiple neurotrophic factors, and/or directly by remyelination. This was supported by evidence that the same hiPSC-derived neurospheres transplanted into the injured spinal cord of NOD/SCID mice, which have increased immunosuppression compared with athymic nude rats, survived for at least 56 days, secreted neurotrophic factors, enhanced angiogenesis, and formed synaptic connections with host axons during remyelination, resulting in the promotion of axonal regrowth and functional recovery^33^. In the future, if HLA-matched hiPSC-derived neurospheres and nerve conduits are clinically and successfully transplanted into peripheral nerve defects of patients, they will survive for longer, and more efficient and successful axonal regeneration will occur^56^.

There are some limitations to the present study. We used the term Schwann-like because it has not been proven that hiPSC-derived Schwann-like cells possess the same properties and specific RNA expression in vitro as native Schwann cells. The outcomes of this study are limited to 5 mm small size nerve defects in rats and it should be evaluated in longer defects in further studies. Some rats died during the follow-up period. Axonal regeneration was evaluated histologically only on cross-sections, not longitudinal sections, at the midpoint of the nerve repair site by non-blinded observers. In the functional recovery assessment, the control group with intact sciatic nerve was absent. With regard to the potential mechanism of action of transplanted cells, grafted iPSc-derived Schwann-like cells could be indirectly responsible as supportive cells for the release of some kinds of growth factors (e.g., nerve growth factor, fibroblast growth factor, brain-derived neurotrophic factor, and glial cell line-derived neurotrophic factor), probably resulting in the enhanced recruitment of endogenous Schwann cells and macrophages and clearance of myelin debris or direct reformation of the myelin sheath^27,53,59,60^. It is unclear which molecules and signaling pathways are involved in the process of promoting nerve regeneration. Further studies are necessary to elucidate the exact mechanisms by which iPSc-derived Schwann-like cells promote the regeneration of peripheral nerves.

## Conclusions

Nerve conduits three-dimensionally coated with the hiPSC-derived neurospheres containing neural stem/progenitor cells promoted axon regeneration and myelination histologically and improved functional recovery for the treatment of sciatic nerve defects in immunosuppressed rats. Therefore, transplantation of the hiPSC-derived neurospheres with nerve conduits is a promising clinical iPSC-based cell therapy for the treatment of peripheral nerve defects.
